# A Novel High-Sensitivity, Low-Power, Liquid Crystal Temperature Sensor

**DOI:** 10.3390/s140406571

**Published:** 2014-04-09

**Authors:** José Francisco Algorri, Virginia Urruchi, Noureddine Bennis, José Manuel Sánchez-Pena

**Affiliations:** 1 Display and Photonic Applications Group, Electronic Technology Department, Carlos III University, Butarque 15, 28911 Leganés, Madrid, Spain; E-Mails: vurruchi@ing.uc3m.es (V.U.); jmpena@ing.uc3m.es (J.M.S.-P.); 2 CEMDATIC, ETSI, Polytechnic University of Madrid, Ciudad Universitaria, 28040 Madrid, Spain; 3 Institute of Applied Physics, Military University of Technology, Kaliskiego 2, 00-908 Warsaw, Poland; E-Mail: nbennis@wat.edu.pl

**Keywords:** temperature sensors, liquid crystals, microstructure

## Abstract

A novel temperature sensor based on nematic liquid crystal permittivity as a sensing magnitude, is presented. This sensor consists of a specific micrometric structure that gives considerable advantages from other previous related liquid crystal (LC) sensors. The analytical study reveals that permittivity change with temperature is introduced in a hyperbolic cosine function, increasing the sensitivity term considerably. The experimental data has been obtained for ranges from −6 °C to 100 °C. Despite this, following the LC datasheet, theoretical ranges from −40 °C to 109 °C could be achieved. These results have revealed maximum sensitivities of 33 mV_rms_/°C for certain temperature ranges; three times more than of most silicon temperature sensors. As it was predicted by the analytical study, the micrometric size of the proposed structure produces a high output voltage. Moreover the voltage's sensitivity to temperature response can be controlled by the applied voltage. This response allows temperature measurements to be carried out without any amplification or conditioning circuitry, with very low power consumption.

## 1. Introduction

Over time temperature monitoring has been an indispensable tool for sensing changes in many different processes and systems, in both research and industrial applications. Many diverse sectors, such as transport, control, automobile, industrial machinery, energy, medical, food, *etc.*, are profiting from the incorporation of temperature sensor equipments. Currently, the temperature sensors range is broad enough to categorize them according to different criteria; the main one is contact and non-contact sensors (pyrometers). Nevertheless, the temperature dependent magnitude (sensing magnitude) and the inherent manufacturing technology are the rules mainly considered to classify them. In some way, this classification estimates the global extent of the conditioning and measurement circuits on the electrical design. The sensing magnitudes are usually connected to the output signals by a relationship derived from an interrogating circuit. Those signals often are in the electrical domain, such as a voltage, a current or a resistance; other times they are in the optical domain, for example, an optical transmittance or a reflectivity. Conventional contact temperature sensors can be either metallic (resistance temperature sensors, thermocouples, bimetallic structures) or semiconductor (thermistor, diode, chip integrated circuit)-based transducers. The highest sensitivities are typical of the semiconductor sensors with relative sensitivities ten times greater than those of metallic sensors [1]. However, semiconductor thermistors are known to have a strong nonlinear relationship between temperature and resistance/voltage outputs, but they are used to more accurately measure the output signals. In particular thermocouples exhibit a high linearity over a wide temperature range. In the last years one of the most used temperature sensors is the chip integrated circuit. This sensor, based on integrated transistors, generates higher output than thermocouples, is more accurate than thermistors and is completely linear. As the circuitry is sealed it is not subject to oxidation. The main disadvantages are the self-heating and the slow response. On the other hand, pyrometers use the radiation from very hot objects as a sensing magnitude through non-contact measurements.

An alternative technology for temperature sensing is the use of liquid crystals (LCs). Some kinds of liquid crystals are thermotropic, that is, they exhibit a set of phase transitions as temperature changes. In addition, LCs show intrinsic anisotropy for some properties (refractive index, permittivity, *etc*.) in the range between the melting (defined as the temperature of melting from solid state into a LC) and the clearing (defined as the temperature at which a LC converts to an isotropic liquid) temperatures. These two features combined allow LCs to be used for temperature sensing in many environments. In particular, LC devices stand out because of their low weight, cost, and power consumption. Liquid crystals are insensitive to any other property likely to be encountered in the device environment such as electromagnetic interferences. Furthermore, they also lack mobile parts which represents a significant advantage when a magnitude is tuned for a specific application. There are not many studies in the literature that take advantage of the properties of LCs The most broad range LC temperature sensors are based on cholesterics [2,3]. These LCs have a helical configuration that results in a material with a high light rotatory power. This fact is their main operation principle; for wavelengths λ = *n*·*p*, where *n* is refractive index and *p* the helical pitch, the light is selectively reflected. This sensor takes advantage of the temperature dependence of the LC helical pitch as a sensing magnitude. The output parameter is the reflected wavelength when a white light strikes the sensor. Temperature-color transducers are usually manufactured on flexible substrates [4]. These kinds of sensors are cheap and easy to measure by means of a fiber optic link. They have been proposed for use in medical applications [5], food processing [6], *etc*.

Despite the fact cholesteric temperature sensors are the most common approach, in the last few years some attempts employing nematic liquid crystals have been carried out. Most of these sensors are based on optical properties of a nematic LC and have the LC refraction index, n, as the sensing magnitude. When the LC is introduced in some specific structures such as Fabry-Perot cavities, or photonic crystal fibers (PCF) [7,8], lambda shifts result as the output signal. In the first case, sensitivities around 1 nm/°C are obtained for maximum temperatures of 65 °C [9]. For the case of PCF, some works have demonstrated sensitivities of 0.22 nm/°C [10], −3.8 nm/°C [11,12] and even 54 nm/°C but for very small temperature ranges (34 °C to 35.5 °C) [13]. These systems are complex to build (filling the PCF is not an easy task) and require complex interrogating circuitries (a spectrum analyzer is usually required). Other approaches, based on the same sensing parameter, produce intensity variations on light passing through the sensor, as the output signal. For example, measuring the transmitted light in structures with plasmonic particles [14] or filling waveguides in ring microresonators [15]. In summary, all of these systems have small temperature ranges and the sensitivities are usually low.

**Figure 1.**
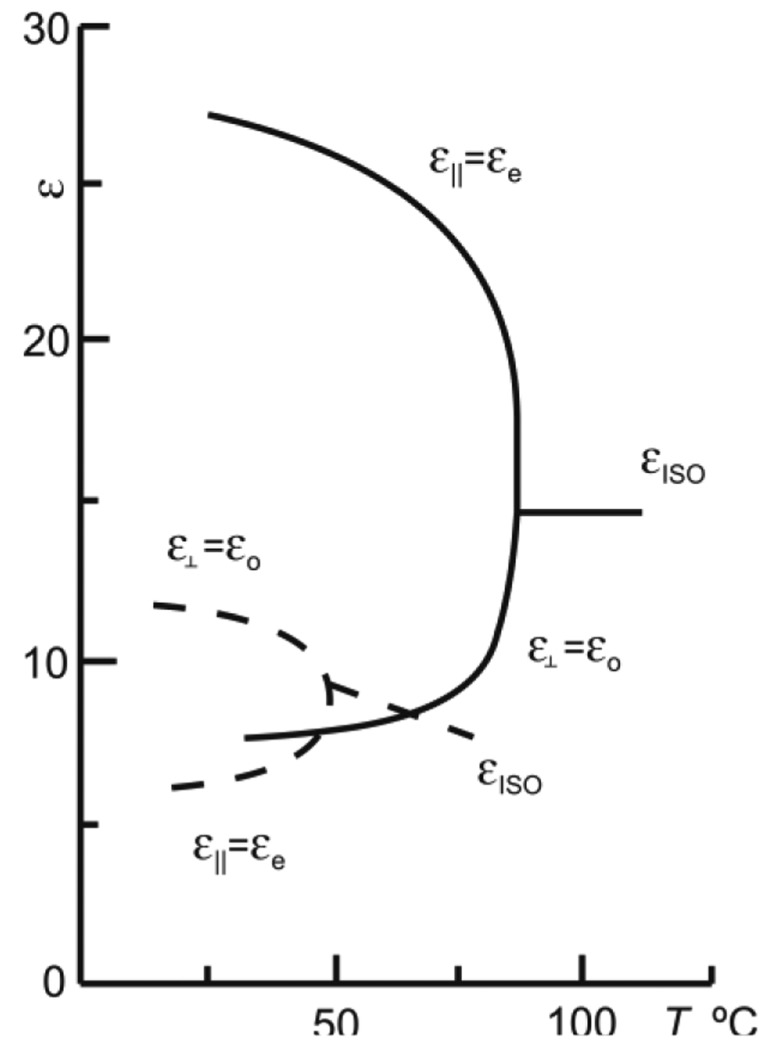
Typical behavior of the real permittivity temperature dependence for two nematic liquid crystals (LCs) with positive (solid line) and negative (dashed line) permittivities [13]. With kind permission from Springer Science+Business Media B.V.

The parameter used to determine the LC interaction with electrical signals is often expressed by permittivity, ε, (related in a quadratic proportion to refractive index). The absolute permittivity means the resistance encountered by an electrical field in a determined medium, that is, the material ability to transmit an electrical field. This parameter is also temperature dependent on as shown in Figure 1 [16].

Very few works can be found in the literature that exploit the permittivity as a sensing magnitude. A nematic LC sensor based on the LC electrical properties was first reported in 2012 [17]. This system generates a variable frequency as an output signal; the result is a temperature-frequency transducer. This kind of sensor has been demonstrated to have broad temperature range. Some disadvantages are that it also needs an interrogating circuit to measure the signal and the resulting sensitivity is directly proportional to the permittivity change with temperature, by a relationship derived from the interrogating circuit.

LC permittivity analysis is frequently carried out by electrochemical impedance spectroscopy (EIS). There are reported some studies that show the growing interest in the characterization of LC mixtures by this method [18,19]. EIS technique measures the frequency dependence of the impedance of a medium, and usually gives the result in a Bode diagram. Measured impedances are related to the components of an equivalent electrical circuit (EEC). Particularly, the electrical components are directly linked to the capacitance of the empty cell, *C*_0_ (the capacitance of the sample when it is filled with air), by the permittivity.

In this work, a novel LC temperature-voltage transducer is proposed. This sensor takes advantage of the temperature dependence of the LC permittivity as the sensing magnitude. For this, a micrometer structure based on tin-doped indium oxide (ITO) interdigitated comb electrodes, acts as an integrated interrogating circuitry. This results in a high voltage output that does not need any type of amplification circuitry. The high impedance of the LC produces very low power consumption (∼μW). In consequence, the self-heating is negligible. The EEC of the LC is studied by EIS to determine the governing electrical equations of the sensor. The EEC of the LC in combination with the proposed structure produces a distributed impedance divider. The analytical study reveals that permittivity change with temperature is introduced in a hyperbolic cosine function, increasing the sensitivity term considerably. The voltage response, linearity and sensitivity for certain supply voltages, and its low power consumption, improves the characteristics of other previous related LC sensors and even of some commercial silicon based sensors (20 mV/°C, double that of most silicon temperature sensors). Moreover, a good stability and no hysteresis have been observed in the experimental measurements.

## 2. Structure and Experimental Set-Up

### 2.1. Structure

**Figure 2.**
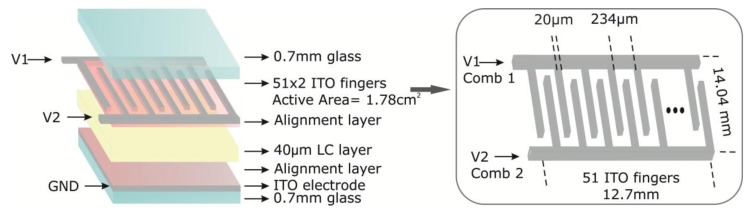
Liquid crystal temperature sensor. (**a**) Three dimensional (3D) diagram and (**b**) Detail of the interdigitated electrode pattern.

The LC sensor structure is similar to a conventional LC display, that is, a sandwich configuration with the material confined between the control and ground plane electrodes (Figure 2). The control electrode consists of a pair of comb-type finger electrodes. One comb electrode, comb 1, is supplied by an AC (Alternating Current) voltage source (V_1_); the other electrode, comb 2, is used for measuring a voltage drop that senses the temperature dependence of the sensor. This arrangement makes the sensor active area dependent on the number of fingers of the electrodes. The electrode pattern is made on ITO coated glass substrates which are etched with a special chrome mask by high resolution photolithography. The glasses are 0.7 mm thick and the ITO layer has a very low resistance sheet (20–50 Ω/sq). Each comb of electrodes has 51 fingers with a distance between them of *r* = 107 μm, forming the 102 fingers an active area of 1.78 cm^2^. In order to have a molecular order inside the cell, a process to obtain a homogeneous alignment has been carried out. In this way, an organic polymeric material (polyimide) is deposited in each glass surface, the glass is spun at 4500 rpm (20 s) and afterwards cured in an oven at 150 °C (100 min). In the last step of this process, the polyimide is rubbed using an in-house machine with velvet covering a rubbing wheel. This causes nearly parallel microgrooves in a common direction; when a cell is filled with a nematic LC, the molecules align nearly parallel to the glasses. Possible ions caused by the process are removed by using a deionizer air gun. The nematic LC MDA 98-1602, from Merck (White House Station, NJ, USA), has been chosen for filling the sensors. The LC parameters are taken from datasheet issued from Merck. The dielectric anisotropy is Δε = 12; the real extraordinary and ordinary permittivities are, ε′_e_ = 16.2 and ε′_o_ = 4.3, respectively. The imaginary part of the permittivity (ε″) is not provided. The clearing point is 109 °C and the low temperature storage −40 °C.

### 2.2. Experimental Set-Up

**Figure 3.**
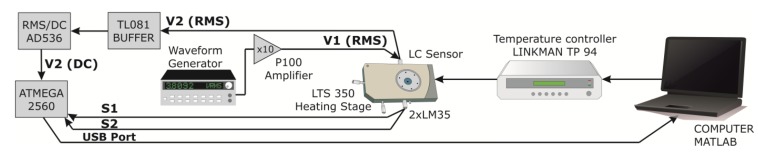
Experimental set-up for characterization of the LC temperature sensor.

The experimental set-up is based on a LTS350 heating and freezing stage handled by a TP 94 system controller (Linkam Scientific Instruments Ltd., Tadworth, UK). For better accuracy, two additional precision centigrade temperature sensors have been added to the system. These redundant sensors avoid possible losses in calibration of the controller system and take into account possible temperature gradients caused by the lack of thickness homogeneity of the LC sensor. This gradient could be avoided with the use of thinner glasses (0.175 mm, for example). The complete set-up (Figure 3) is controlled by an automated MATLAB programmed for this purpose. This program processes the measured data and directly show some graphs of temperature dependence of voltage.

The heating stage increases the temperature at a rate of 1 °C per minute. The source of supply is an AC voltage controlled via a HP 33120A waveform generator (Agilent Technologies, Santa Clara, CA, USA) and connected to V_1_, through a high voltage broadband amplifier (P100). The output is 10 times the input voltage. AC square voltage signals have been chosen and no more than 50 V_rms_ can be generated considering the limited voltage output (±50 V). As commented before, two LM35 temperature sensors measure the temperature, *T*, inside the stage. These silicon sensors are placed in each LC sensor surface and the outputs (S1 and S2) are connected to two analog inputs of ATMEGA 2560 microcontroller (Atmel Corporation, San Jose, CA, USA). This one sends data through USB port, and then data are processed by a MATLAB program. Three samples per minute are taken. The temperature changes the permittivity values, and consequently the voltage at V_2_. This voltage is connected to a TL081-based buffer. The output of this buffer is converted to a DC (Direct Current) signal by an AD536 RMS/DC converter (Analog Devices, Norwood, MA, USA). This chip converts the RMS (root mean square) value to the same absolute value in DC voltage (for example, 1 V_rms_ to 1 V_dc_). The output of this chip is also connected to microcontroller's analog input and the measured data is stored by MATLAB.

## 3. Results and Discussion

### 3.1. Modeling of the Equivalent Electric Circuit

**Figure 4.**
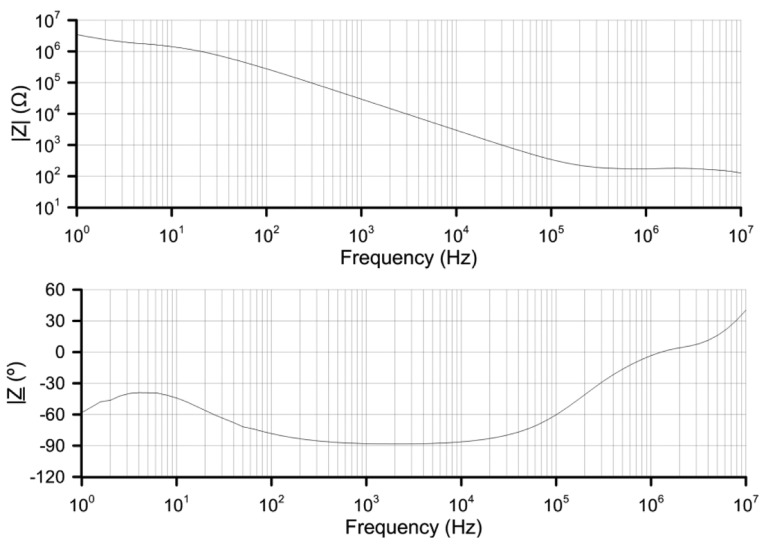
Frequency response of MDA 98-1602 nematic LC given as (**a**) impedance module and (**b**) phase.

The response of normal materials to external fields generally depends on the frequency of the field. In the case of nematic LC this permittivity is complex due to material losses. In order to develop an electrical model of the proposed sensor, the EEC of the LC is essential; this one has been measured by EIS. A Solartron 1260 Impedance/Gain-Phase Analyzer (Solartron Analytical, Hampshire GU14 0NR, UK) been used to measure a sample monopixel cell of 8 cm^2^ and thickness of 6 μm. The results for a supplied AC voltage of 50 mV_rms_ are shown in Figure 4.

Looking at the phase graph, from 500 Hz to 5 kHz the phase shift is almost −90° (minimum −88°). This indicates a capacitive behavior with some losses that is usually modeled as a capacitor in parallel with a resistance. Taken into account the capacitance of the empty cell *C*_0_ = 1.18 nF, and Equation 1:

(1) $${ɛ}^{*}=\frac{1}{i\omega C_{0}Z^{*}}=ɛ\prime +iɛ\prime \prime =\frac{Z^{\prime \prime }}{(Z^{\prime 2}+Z^{\prime \prime 2})\omega C_{0}}+\frac{iZ^{\prime }}{(Z^{\prime 2}+Z^{\prime \prime 2})\omega C_{0}}$$

The real ordinary permittivity is 4.5 in this frequency range. The imaginary part is minimal from 1 kHz to 5 kHz (0.15). From Equation (2) the components of the EEC, for this range of frequencies, can be estimated:

(2) $$\begin{array}{cc} C=\frac{{ɛ}_{0}ɛ\prime S}{d}, & R=\frac{d}{{ɛ}_{0}ɛ\prime \prime \omega S} \end{array}$$

**Figure 5.**
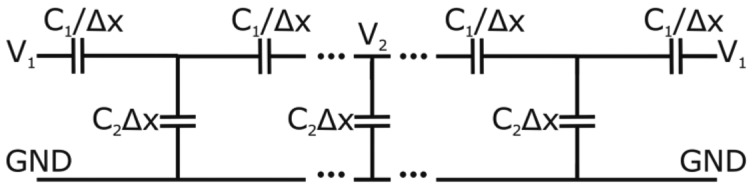
Simplified EEC of the LC sensor.

In this range of frequencies, where the imaginary part is minimal, the parallel resistance is very high. For this reason, we conclude that for 1–5 kHz this nematic LC can be considered as a simple capacitor. For higher frequencies there is a dramatic decline in the resistance value (*R*) so this term cannot be neglected. When this LC is introduced in the proposed structure, it results in a distributed impedance divider, composed of distributed capacitors. In Figure 5 a simple EEC model for the proposed structure is shown.

The distributed capacitor, *C*_1_, depend on the permittivity in the parallel direction (with respect to the substrates), and *C*_2_ depends on the permittivity on the perpendicular direction. The voltage gradient, caused by the impedance divider, depends strongly on the permittivity parameters of the EEC components. Using the coplanar theory [20] and the classical transmission line theory the resulting governing equation for the structure is:

(3) $$\frac{\partial V^{2}(x)}{\partial x^{2}}=\frac{C_{2}}{C_{1}}\cdot V(x)$$

The components of the model are defined per unit length, Δ*x* (*x*-axis is the direction of the transmission line) and determined by:

(4) $$\begin{array}{cc} C_{1}=\frac{{ɛ}_{0}\cdot ɛ\prime_{1}\cdot d}{A}, & C_{2}=\frac{{ɛ}_{0}\cdot ɛ\prime_{2}}{d} \end{array}$$

where constant *A* can be estimated using conformal mapping based on the Christoffel–Schwarz transformation (2.5 in this case), *d* is the LC thickness, ε_0_ the vacuum permittivity, and ε′_1_ and ε′_2_ the average real effective permittivities in the horizontal and vertical directions, respectively. The solution to Equation (1), considering the voltages on comb 1, V_(x = −r)_ = V_(x = r)_ = V_1_, the voltage on comb 2, V_(x = 0)_ = V_2_, and considering Equation (4), is:

(5) $$V_{2}=\frac{V_{1}}{\cosh\left(\frac{r}{d}\sqrt{\frac{{{ɛ}^{\prime }}_{2}A}{{{ɛ}^{\prime }}_{1}}}\right)}$$

This equation reveals a direct effect of the relation between electrode distance (*r*) and sample thickness (*d*) on the output voltage. From Equation (5) an approximation to the voltage output can be estimated and the structural parameters optimized. At 25 °C (the permittivities are given for this temperature in the datasheet) and for a molecular position where the relation between permittivities is 6/14 = 0.4 (a typical case in which some of the molecules are tilted, low voltages), V_2_ = 0.1 · V_1_. As commented before, LC has a high permittivity anisotropy that can be controlled by voltage. This fact causes that ε′_1_ differs from ε′_2_ as a function of voltage. These two parameters are temperature dependent, with differences in the dependence with temperature between ε′·and·ε′_e_. This results in a theoretical control in sensor sensitivity with voltage, *S* = ΔV_2_/Δ*T* (mV/°C). The suppression of ε′_1_ parameter could increase the sensitivity considerably. A possible solution would be the inclusion of some material in the LC surface that does not change with temperature but distribute the voltage (a resistivity layer, for example [21]).

### 3.2. Frequency Study

**Figure 6.**
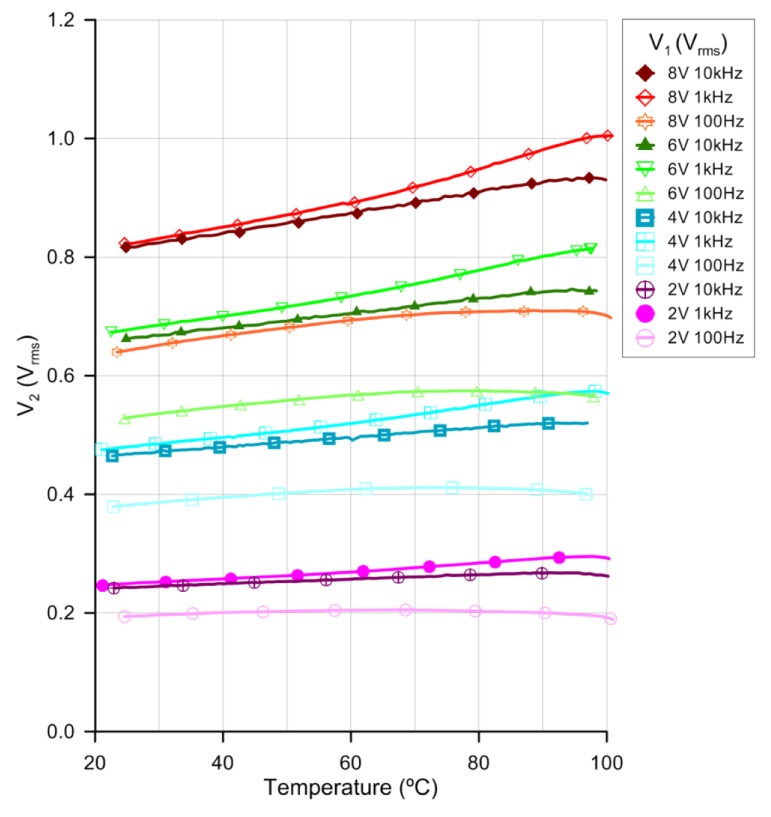
Temperature dependence of the LC sensor response for several voltages and frequencies at V_1_.

In order to find the optimum operation frequency, a sweep frequency study has been performed. Three representative frequencies, for four different voltages, are displayed in Figure 6 in a temperature range between 20 °C and 100 °C (below the clearing temperature).

Due to the large number of samples only some measurements are identified by symbols. All the measurements have been done by lowering and raising the temperature. No differences on the offset error have been observed between both experiments, concluding that the device has not hysteresis. The linearity of the response differs as a function of the supply voltage frequency. For example for V_1_ = 4 V_rms_ the linear regression coefficients (*R*^2^) are 38%, 99.4% and 99.3% for frequencies of 100 Hz, 1 kHz and 10 kHz, respectively. Furthermore, for V_1_ = 8 V_rms_, *R*^2^ is 86%, 98.8% and 99.6% for frequencies of 100 Hz, 1 kHz and 10 kHz, respectively. The best linearity is observed for 1 kHz and 10 kHz. From the sensitivity point of view, these results have shown an optimum behavior at around 1 kHz. Looking at Figure 4, we can observe the most capacitive behavior at this frequency. For 1 kHz the ε′ is predominating so, one reason for this behavior could be different changes of the value of the ε′ with temperature than the ε″. On the other hand, an increase in the voltage's sensitivity to temperature is observed for greater voltages. Following the reasoning of the previous argument, this is probably caused by greater changes with temperature of ε′_e_ than ε′_o_ (as it was observed in Figure 1). This study leads us to a broad range temperature measure at 1 kHz voltage frequency for different voltages.

### 3.3. Voltage Study

**Figure 7.**
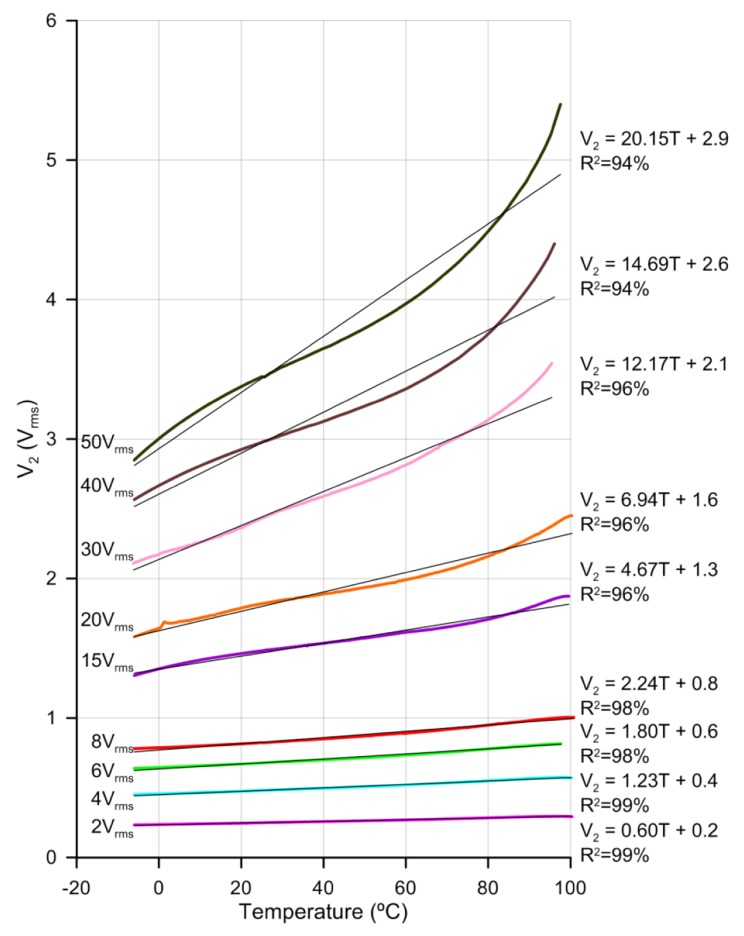
Temperature dependence of the LC sensor response for several voltages at V_1_. Linear fits indicate the approximate sensitivity and R^2^.

As noted above, the higher the voltage the higher the sensor sensitivity. In Figure 7 the voltage, V_1_, has been increased from 2 V_rms_ to 50 V_rms_, in the temperature range from −6 °C to 100 °C, approximately.

**Figure 8.**
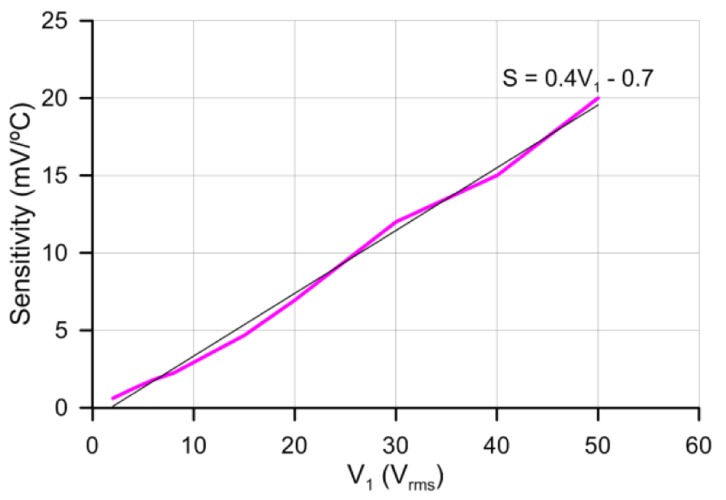
Sensitivity as a function of supply voltage (V_1_) and linear fit.

As with previous results, no hysteresis has been observed. There is an increase of the sensitivity with voltage but at the cost of losing linearity. For low voltages the linear regression coefficients are 99%. For higher voltages curves are fitted to a linear curve for comparison purposes (there is an almost linear relation between sensitivity and voltage). Despite this, the non-linear curves can be easily fitted to a three grade polynomial. The relation between voltage and sensitivity is depicted in Figure 8. This relation shows a clear control over sensitivity with voltage.

The graph reveals a slope of 0.4 (mV/°C/V_rms_). Considering the complete temperature range (−6 °C to 100 °C), the maximum output voltage's sensitivity to temperature, is an unusually high 20 mV/°C; approximately double that of most silicon temperature sensors. Probably the application of higher voltages could increase more the sensitivity, as the linear graph indicates. This limit has to be further investigated by using amplifiers of high voltage output (the P100 is limited to ±50 V). Besides, higher sensitivities could be obtained for certain specified ranges.

**Figure 9.**
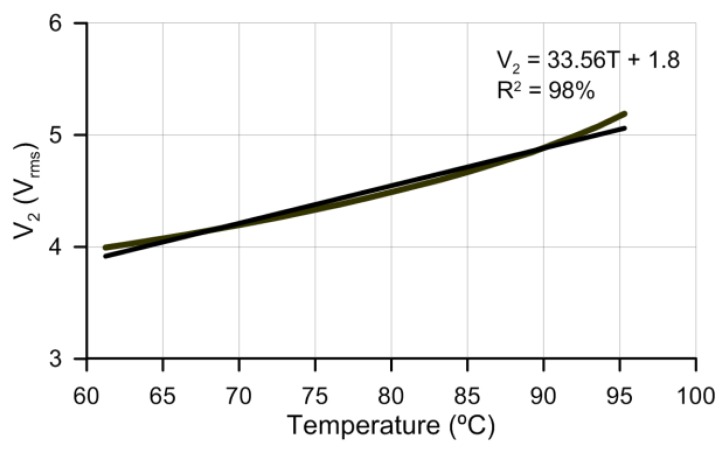
Voltage versus temperature in a range from 60 °C to 95 °C and 50 V_rms_ supply voltage. Linear fit with a 33.5 mV/°C slope.

As shown in Figure 9, in this case, a sensitivity of 33.56 mV/°C is obtained for a temperature range from 60 °C to 95 °C. This range could be useful in some specific applications. For example, LCD displays are used in extreme environments (outdoor, avionics display, military aerospace display). The LCDs fabricated for these kinds of applications usually have operation limits from 60 °C to 85 °C. Working in this range could cause it not to run at all or a severely restricting performance. The sensor could follow the temperature with high sensitivities in this specific range.

Considering the same supply voltage (50 V_rms_), if a range from 5 °C to 35 °C is taken, a sensitivity of 15 mV/°C is obtained. This is the range limit of normal operation in LCD projectors, with the advantage of easy integration due to the similarity of these technologies. A determinant conclusion can be extracted from these results. The high sensitivity obtained for high voltages could be obtained with low voltages if the molecules are placed in perpendicular position in the fabrication process. The use of another type of alignment layer, as SiO*_x_* [22] for example, could be a solution. This type of alignment layer determine the anchoring strength, nematic LC molecules are aligned perpendicularly to the substrate when the device is filled. This idea has to be further investigated but probably the sensitivity would be maximal with low voltages.

### 3.4. Power Consumption Study

**Figure 10.**
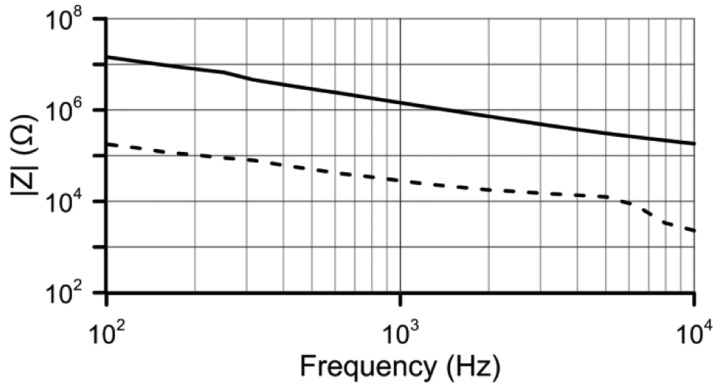
LC sensor impedance graph in a range from 100 Hz to 10 kHz. With 2 V_rms_ of supply voltage (solid line) and 30 V_rms_ (dashed line).

Another important characteristic is the low power consumption that depends on the cell impedance at the applied voltage. The sensor impedance has been measured in the frequency range from 100 Hz to 1 kHz (Figure 10). Because of impedance meter limitations the high voltage measurement is done for 30 V_rms_ at V_1_.

Considering that the optimum frequency is 1 kHz and P = V^2^/|Z|, the maximum and minimum power consumption is 18 mA–4 μA. When no high sensitivities will be required, this very low supply current makes it ideal for portable equipment and is low enough to make self-heating effects negligible.

## 4. Conclusions

In this work a novel idea in the research field of temperature sensors has been presented. The sensor response has been completely characterized. This new LC temperature sensor has several advantages with respect to other previous related LC sensors. In some aspects, this sensor is an improvement on the characteristics of some commercial temperature sensors. For example, it has higher sensitivities than silicon sensors (for certain supply voltages). In addition, it has more linearity than thermocouple sensors. The problem of thermo-couples or RTD with self-heating is avoided due to its low consumption. There is no need for any amplification due to its high output voltage. The structure is simple and has an active area easy to scale. Coplanar electrodes can be easily patterned at very small dimensions yielding miniaturized, reproducible, and ultimately low cost devices. An increase in sensitivity has been observed with the alignment process. The sensitivity can be adjusted with voltage depending on the selected application.

As commented before, the voltage output could be improved with structure designs. An in-depth investigation of the EEC will lead us to optimum designs. The incorporation of other materials to this structure is also being studied in order to improve the sensor characteristics (a resistivity layer can improve the sensitivity). The inclusion of a special alignment layer that results in a perpendicular alignment of the LC molecules could result in high sensitivities for low voltages. These ideas have to be further investigated. This sensor can be used in LCD displays, LCD projectors, portable equipments or any application where its properties procure an advantage with respect to current available sensors. This work has presented and characterized a novel idea, and opened new avenues for research.
